# FePO_4_ nanoparticles produced by an industrially scalable continuous-flow method are an available form of P and Fe for cucumber and maize plants

**DOI:** 10.1038/s41598-019-47492-y

**Published:** 2019-08-02

**Authors:** Davide Sega, Giuseppe Ciuffreda, Gino Mariotto, Barbara Baldan, Anita Zamboni, Zeno Varanini

**Affiliations:** 1 Department of Biotechnology, University of Verona, Verona, Italy; 2 Fabbrica Cooperativa Perfosfati Cerea, San Pietro di Morubio, VR Italy; 3 Department of Computer Science, University of Verona, Verona, Italy; 4 Department of Biology, University of Padua, Padua, Italy

**Keywords:** Plant physiology, Design, synthesis and processing

## Abstract

Nanomaterials are widely used in medical and pharmaceutical fields, but their application in plant nutrition is at its infancy. Phosphorous (P) and iron (Fe) are essential mineral nutrients limiting in a wide range of conditions the yield of crops. Phosphate and Fe fertilizers to-date on the market display low efficiency (P fertilizers) and low persistence in soil (Fe fertilizers) and negatively affect the environment. In the tentative to overcome these problems, we developed a continuous industrially scalable method to produce FePO_4_ NPs based on the rapid mixing of salt solutions in a mixing chamber. The process, that included the addition of citrate as capping agent allowed to obtain a stable suspension of NPs over the time. The NPs were tested for their effectiveness as P and Fe sources on two hydroponically grown crop species (cucumber and maize) comparing their effects to those exerted by non-nanometric FePO_4_ (bulk FePO_4_). The results showed that FePO_4_ NPs improved the availability of P and Fe, if compared to the non-nano counterpart, as demonstrated by leaf SPAD indexes, fresh biomasses and P and Fe contents in tissues. The results open a new avenue in the application of nanosized material in the field of plant nutrition and fertilization.

## Introduction

High-yield agriculture is strictly dependent on the use of fertilizers. However, their efficiency represents a limit both for the economy and the sustainability of crop production. This is mainly due to the low value of fertilizers Nutrient Use Efficiency (NUE), which is lower than 50% for nitrogen, around 40% for potassium and around 10–20% for phosphorus^1^. For this reason, fertilizers need to be improved, optimizing them for nutrients release and increased availability in order to foster crop production and reduce environmental consequences. In this context as stated by several recent reports^2,3^ nanotechnology is believed to offer a great potential to achieve the goal.

Phosphorous and Fe are essential mineral nutrients limiting in a wide range of conditions the agricultural productivity. Phosphate fertilizers to-date on the market have a very low use efficiency that does not exceed 10–20%. Moreover, the raw material for the production of P fertilizers, stocks of rock phosphates, are running out^4^. Although Fe is present in the soil in high total amounts, it is scarcely available in aerobic soils. Iron fertilizers present on market have a limited temporal effectiveness and are expensive and potentially harmful for the environment^5^. It is estimated that P deficiency occurs in almost 65% of soils^6,7^ while Fe deficiency occurs in about 30% of soils^6^. It is therefore evident the need to develop more efficient fertilizers for these two nutrients^8–13^.

Nanomaterials are widely used in medical and pharmaceutical fields, but examples of their application in plant nutrition are very few and no one described the use of FePO_4_ nanoparticles (FePO_4_ NPs). However, some recent works have demonstrated the promising perspective as mineral nutrient suppliers of nanosized materials containing these elements, mainly focusing on Fe-oxides, Fe-carbonates and Ca-phosphates^14–19^ tested on plants grown in pot or greenhouse. It has been in fact ascertained that the availability of these nutrients can be enhanced by reducing the size of oxides and salts to the nanometric scale. On the other hand, it has been also shown that the same material (*e.g.* Fe-oxide) can have different efficacy, depending on plant species and growth substrate.

Beyond the results, there is however often a lack of adequate controls in many pieces of literature (*e.g.* bulk counterpart, or electrolytic counterpart, or negative control), making difficult the comparison among available data. In order to understand the influence of particles size on nutrient availability, it is in fact important to set adequate control conditions, that help to ascribe observable effects to variables such as size, chemical form, etc^20^.

It is known that plant strategies for Fe and P mobilization share several mechanisms, as proton extrusion and the secretion of carboxylic acids and phenolic compounds^21–25^. For this reason, FePO_4_ could be a promising material, supplying two essential nutrients in a controlled-delivery way taking advantage of common plant responses.

Large-scale industrial production of nanofertilizers is yet to be realized and there is a need to develop ideas and concepts toward process scale-up that could be used by the industry. Many methods were developed for the synthesis and production of nanoparticles (NPs). These methods can be divided in top-down and bottom-up approaches^26^.

Among various chemical methods for synthesis of different types of NPs, the co-precipitation process has several advantages, including low cost, good homogeneity, high purity of product and not requiring organic solvents and heat treatment^27^. For this reason, co-precipitation is widely used for the synthesis of metal oxides NPs^28–30^ but also for the synthesis of NPs made of insoluble salts such as FeCO_3_^18^, Ca_5_(PO_4_)_3_(OH)^19^, CaF_2_^31^ and FePO_4_^32–35^.

Co-precipitation methods are often carried out in batch, as shown in the examples cited above. However, this method can also be carried out in continuous flow, and continuous flow synthesis was demonstrated for FePO_4_ NPs^33–35^. NPs are thermodynamically unstable due to their very high surface energy. For this reason, bare NPs tend to stabilize themselves by lowering the surface area through agglomeration^19,36^, and this can then cause sedimentation over time. In order to avoid it, NPs have to be stabilized^36^. This can be achieved by the use of cappants, stabilizing agents that avoid or minimize the aggregation of NPs. The use of cappants is common when NPs are used in suspension in an aqueous medium^37,38^. However, it is the use of obtained NPs that drives the choice of NPs purification process towards mild methods rather than non-mild ones, and the possible capping. On the other hand, safety of workers is an issue that makes the use of NPs as liquid suspension more desiderable^39^.

Given the lack of industrially scalable methods for producing nano fertilizers, we optimized a simple, economically advantageous and industrially scalable synthesis method for producing FePO_4_ NPs. Furthermore, we evaluated their potential as sources of plant mineral nutrients using two crop species (cucumber and maize), with the adequate experimental controls, in order to discriminate the effects caused by the size, rather than the chemical form.

## Results

### Continuous synthesis with a pilot plant

**Figure 1:**
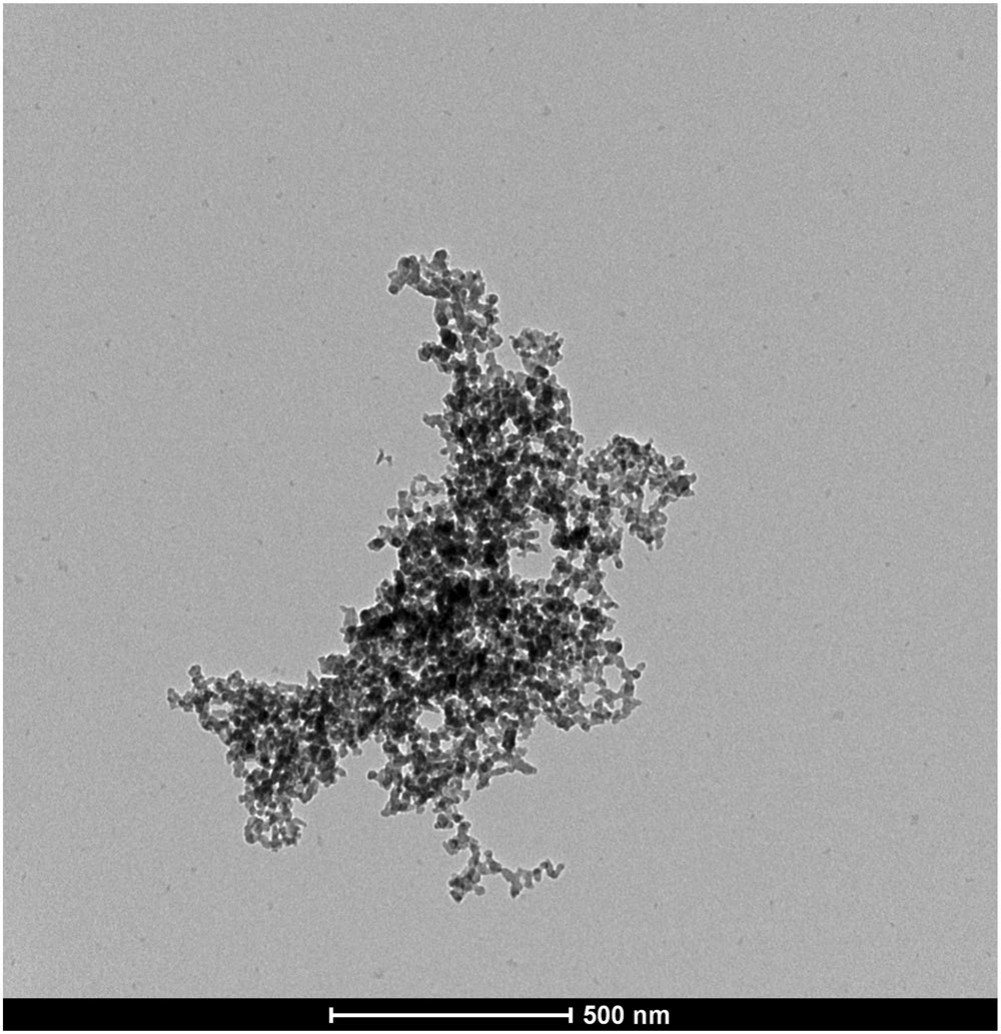
TEM visualization of FePO_4_ NPs obtained with the continuous pilot plant and purified through centrifugation.

**Figure 2:**
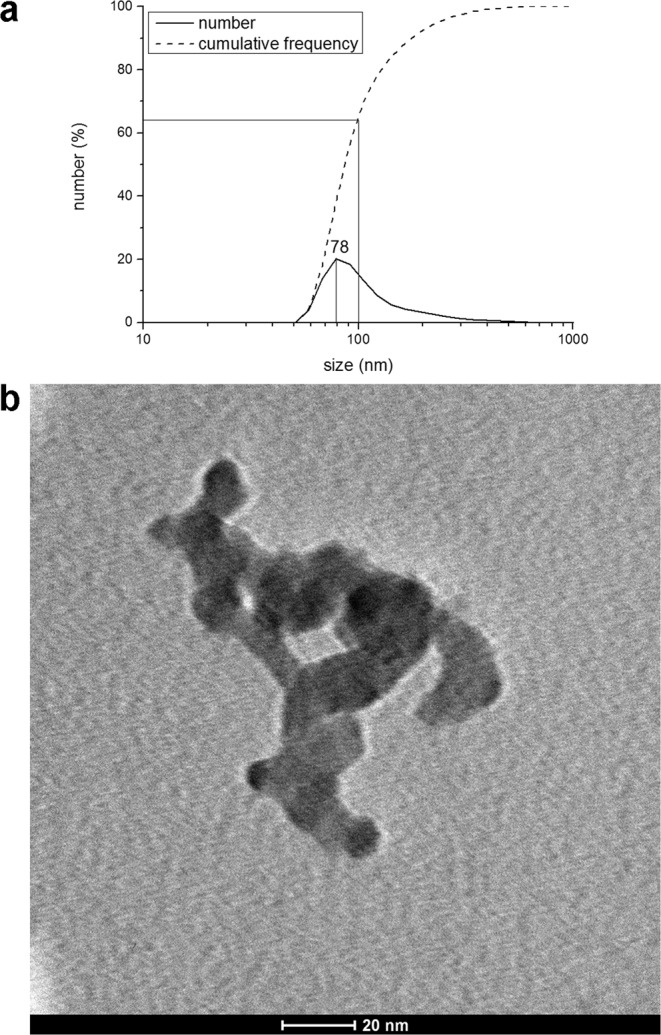
(**a**) Size distribution of FePO_4_ particles obtained with the continuous pilot plant and purified through dialysis. The analysis was performed through DLS technique. (**b**) TEM visualization of FePO_4_ NPs obtained with the continuous pilot plant and purified through dialysis.

A pilot plant for the continuous-flow synthesis of FePO_4_ NPs was set up, after modifications of the method described by Zhang *et al*.^35^. The system could produce 15 L·h^−1^ of raw 0.05 M FePO_4_ NPs suspension, for a productivity of approximately 140 g FePO_4_/h. Two purification methods were considered: centrifugation and dialysis. Transmission Electron Microscopy (TEM) visualization of the obtained FePO_4_ NPs purified through centrifugation is shown in Fig. 1. The so-obtained particles are about 20–25 nm in diameter, but aggregate together to form large aggregates of 500 nm or more. Transmission Electron Microscopy (TEM) visualizations of the obtained FePO_4_ NPs purified through dialysis are shown in Fig. 2b. Also in this case single NPs are much smaller than 100 nm, being about 20–25 nm in diameter, and can aggregate together. However, in the case of purification through dialysis, about 64% of aggregates are smaller than 100 nm, with a peak at 78 nm, as shown by the results of the Dynamic Light Scattering (DLS) analysis (Fig. 2a).

### Bare NPs instability and citrate capping

**Figure 3:**
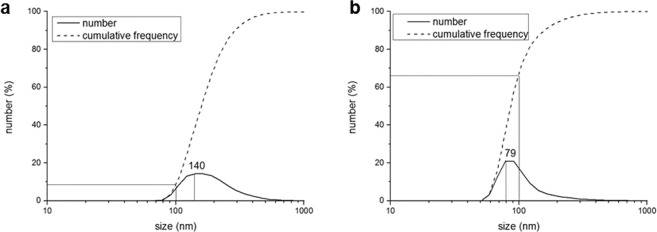
Size distribution of bare FePO_4_ particles (**a**) and citrate-capped FePO_4_ particles (**b**) obtained with the continuous pilot plant after 8 months of storage. The analysis was performed through DLS technique.

FePO_4_ NPs suspensions obtained with the pilot plant, even if small and with more than 50% of the aggregates smaller than 100 nm, showed to be not stable for long period, aggregating and then sedimenting. In fact, comparing the DLS analysis of FePO_4_ NPs suspension after 1 hour of the synthesis (Fig. 2a) with the DLS analysis of the same FePO_4_ NPs suspension after 8 months of storage at room temperature (Fig. 3a), it is possible to see that the peak size shifted from 78 nm to 140 nm, and the portion of aggregates smaller than 100 nm was dramatically reduced from 64% to 8%.

To solve this problem, NPs were citrate capped through the addition of tribasic potassium citrate and thorough vortexing in order to reduce aggregation on long time periods.

The citrate-capping of FePO_4_ NPs is effective (Fig. 3b) in stabilizing the suspension, preventing them from aggregation and sedimentation for at least 8 months.

### Laboratory-scale batch synthesis

Continuous production processes are useful and economically advantageous for making large amounts of NPs. However, these methods are less practical and more wasteful if the production need is limited. For these reasons, a laboratory-scale batch synthesis method was developed for the production of citrate-capped FePO_4_ NPs to be used in our experiments.

**Figure 4:**
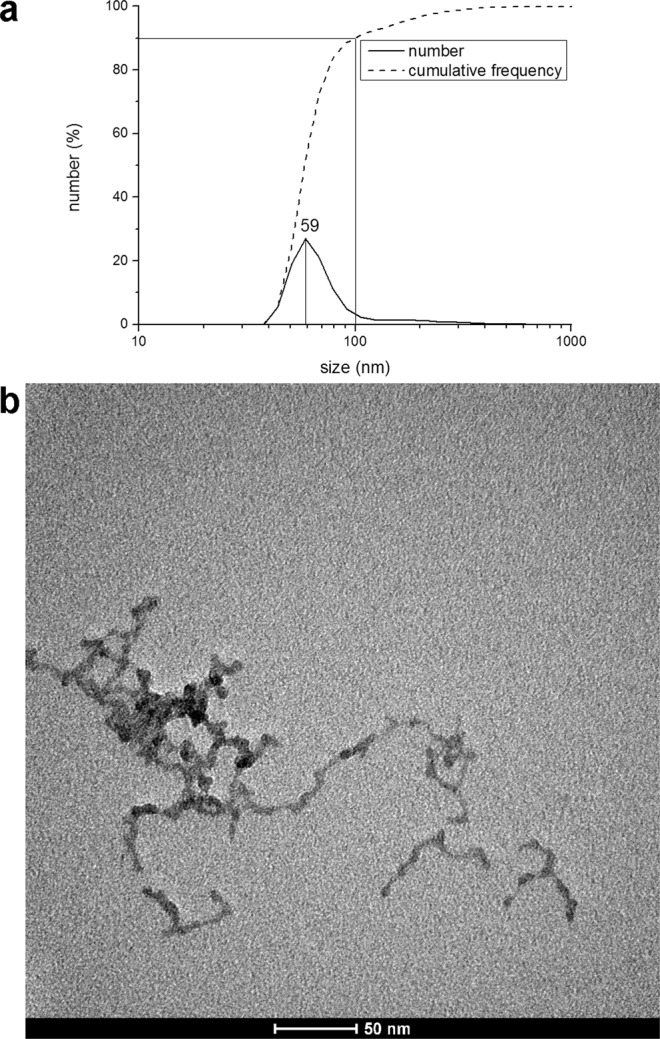
(**a**) DLS analysis of citrate-capped NPs produced with the batch method optimized for laboratory scale. The analysis was performed through DLS technique. (**b**) TEM visualization of citrate-capped FePO_4_ NPs produced with the batch method optimized for laboratory scale.

Figure 4 show the results of DLS analysis and TEM visualization, respectively, of FePO_4_ NPs synthesized at lab-scale. NPs were smaller than 20 nm (Fig. 4b), but could aggregate together, with a size peak of aggregates of 59 nm. About 90% of them are smaller than 100 nm (Fig. 4a). For this sample zeta potential was determined to be −45.0 ± 0.55 mV.

Fe/P molar ratio was 1.055 calculated using the quantification of both elements in the suspension (0.141 M for Fe and 0.133 for P), suggesting the hypothetical formula Fe(PO_4_)_0.95_(OH)_0.15_.

X-Ray Diffraction analysis showed the amorphous nature of FePO_4_ NPs (Supplementary Fig. S1).

### Citrate capping and its role in NPs stabilization

**Figure 5:**
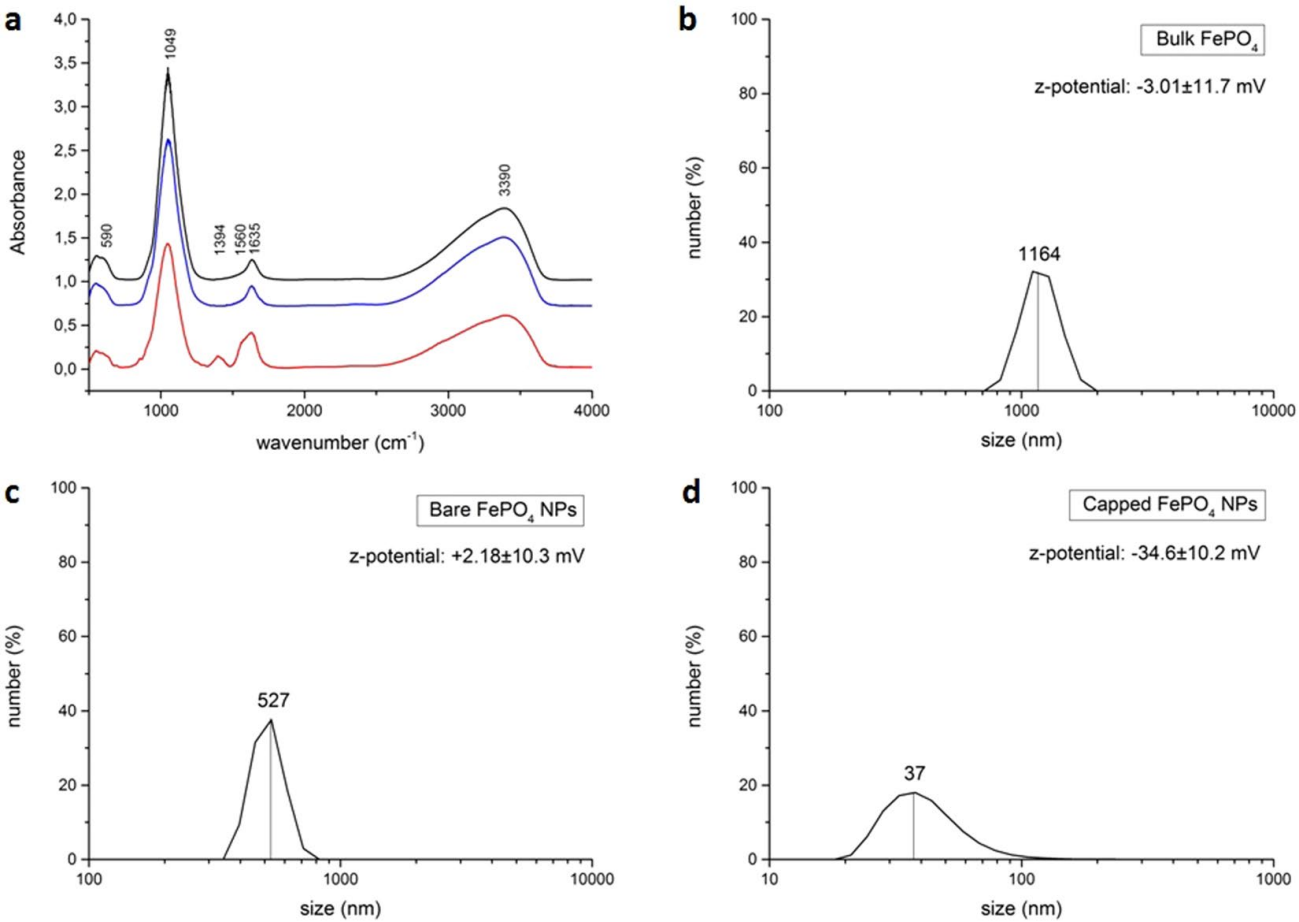
Superimposed FT-IR spectra (**a**) of: bulk FePO_4_ (black line), bare FePO_4_ NPs (blue line) and capped FePO_4_ NPs (red line) and DLS analysis of bulk FePO_4_ (**b**), bare FePO_4_ NPs (**c**), capped FePO_4_ NPs (**d**).

In order to understand if citrate treatment really causes an adsorption of citrate molecules on particles surface, thus modifying their surface chemistry, a batch synthesis was performed and citrate-capped FePO_4_ NPs were compared with bare FePO_4_ NPs and bulk FePO_4_ through FT-IR spectroscopy. In addition, we determined particles surface charge measuring z-potential values and size distribution through DLS. Figure 5a shows the superimposed FT-IR spectra of bulk FePO_4_, bare FePO_4_ NPs and capped FePO_4_ NPs. For all samples the bands at 3390 and 1635 cm^−1^ are ascribed to O-H stretching and H-O-H bending vibrations respectively, indicating the presence of water in the structure^40^. The bands at 1049 and 590 cm^−1^ are ascribed to Fe-O-P and O-P-O stretching vibrations respectively^41–43^. In addition to these bands, we can observe in capped FePO_4_ NPs spectrum the presence of two bands at 1394 and 1560 cm^−1^ that can be ascribed to the symmetrical and anti-symmetrical stretching respectively of C–O in COO^−^ groups, that come from citrate molecules adsorbed on the particles surface^44^. Figure 5c shows that bare FePO_4_ particles have a nearly uncharged surface, as can be seen by z-potential value (+2.18 ± 10.3 mV), therefore forming big aggregates. This is not true for capped FePO_4_ NPs, that thanks to a z-potential of −34.6 ± 10.2 mV, only formed small aggregates of around 37 nm (Fig. 5a).

### Effects of FePO_4_ NPs on plants growth

The obtained FePO_4_ NPs (batch synthesis) were tested for their effectiveness as P and Fe sources in hydroponics on two crop species: cucumber (*Cucumis sativus*), a Strategy I plant, and maize (*Zea mays*) a Strategy II plant for Fe acquisition respectively^45^. The experiments were designed in order to evaluate the effect of FePO_4_ NPs as source of P and Fe. For this reason, as negative controls plants grown without P (-P), without Fe (-Fe), or without both (-P-Fe) were used. In addition, in order to analyse if the size of FePO_4_ particles could cause different effects on plants, we included in the experiment a treatment with non-nanometric FePO_4_ (bulk FePO_4_). Both FePO_4_ NPs and bulk FePO_4_ were added at the same concentration used for P and Fe in the complete nutrient solution (100 µM).

**Figure 6:**
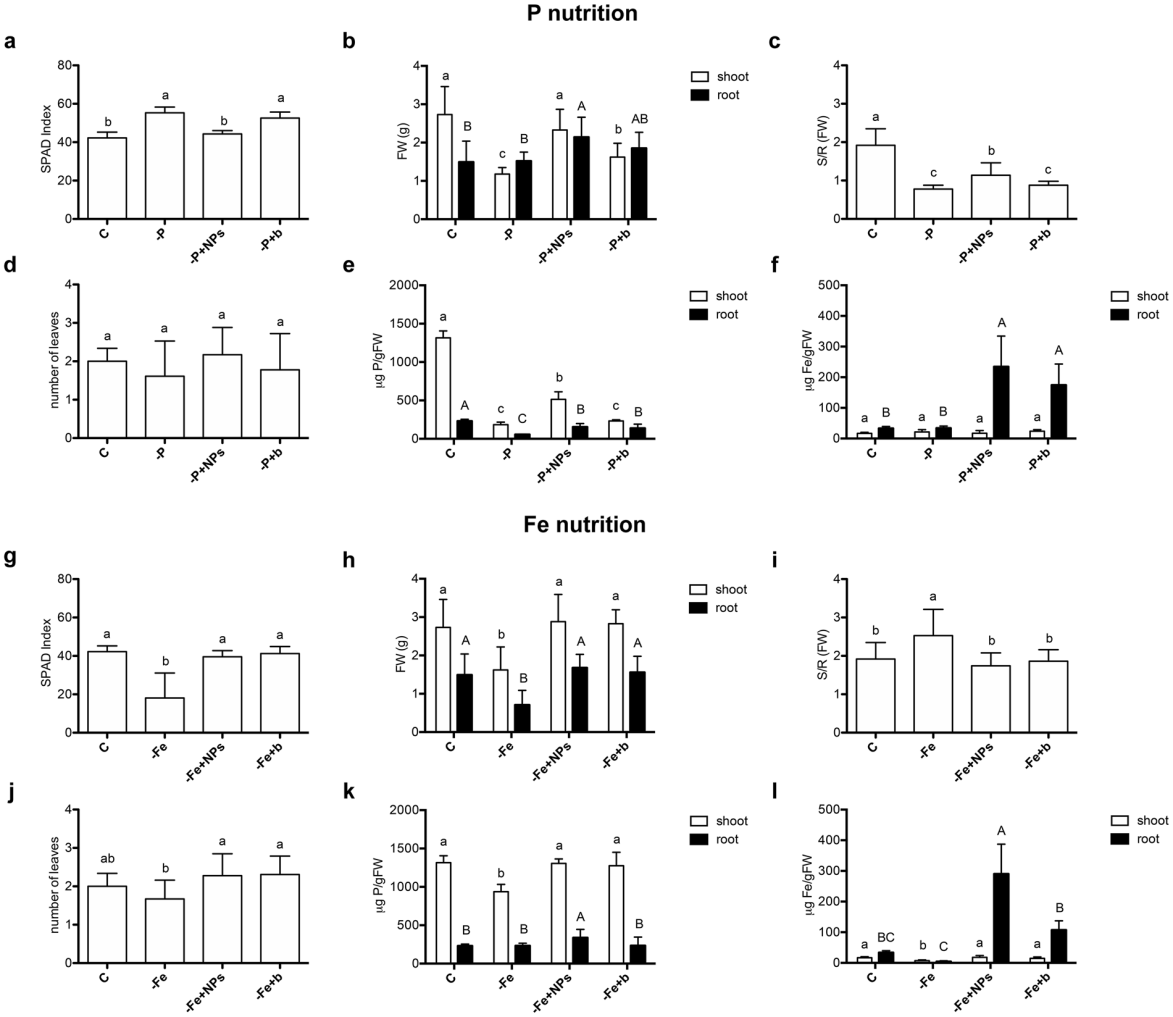
Comparison of cucumber plants treated with different nutrient sources. (**a**–**f**) Control plants (C), plants grown without P (-P), plants grown with FePO_4_ NPs as P source (-P + NPs), plants grown with bulk FePO_4_ as P source (-P + b); (**g**–**l**) control plants (C), plants grown without Fe (-Fe), plants grown with FePO_4_ NPs as Fe source (-Fe + NPs), plants grown with bulk FePO_4_ as Fe source (-Fe + b). SPAD index (**a**,**g**), root and shoot fresh weight (**b**,**h**), shoot/root ratio (**c**,**i**), number of leaves (**d**,**j**), shoot and root P content (**e**,**k**) and shoot and root Fe content (**f**,**l**). Data are means ± SD of three independent experiments with six plants (three plants for **e**, **f**, **k** and **l**) each (one-way Anova with Turkey’s post hoc test, p < 0.05).

**Figure 7:**
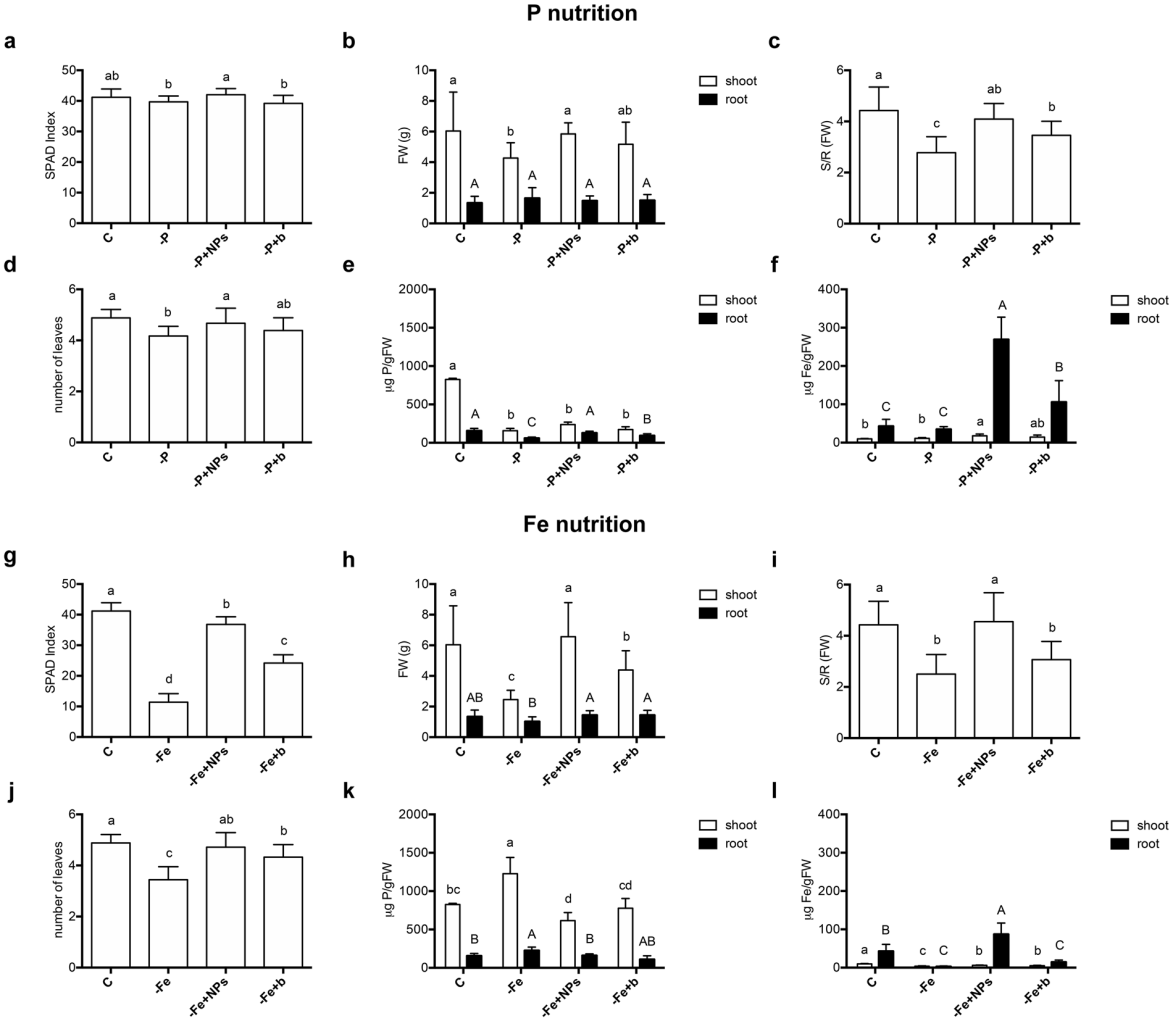
Comparison of maize plants treated with different nutrient sources. (**a**–**f**) Control plants (C), plants grown without P (-P), plants grown with FePO_4_ NPs as P source (-P + NPs), plants grown with bulk FePO_4_ as P source (-P + b); (**g**–**l**) control plants (C), plants grown without Fe (-Fe), plants grown with FePO_4_ NPs as Fe source (-Fe + NPs), plants grown with bulk FePO_4_ as Fe source (-Fe + b). SPAD index (**a**,**g**), root and shoot fresh weight (**b**,**h**), shoot/root ratio (**c**,**i**), number of leaves (**d**,**j**), shoot and root P content (**e**,**k**) and shoot and root Fe content (**f**,**l**). Data are means ± SD of three independent experiments with six plants (three plants for **e**, **f**, **k** and **l**) each (one-way Anova with Turkey’s post hoc test, p < 0.05).

The more meaningful results were observable when evaluating FePO_4_ as source of one nutrient per time (P or Fe) (Figs 6 and 7). Data regarding the double deficiency are reported in Tables S1 and S2. In particular, in Figs 6 and 7 are presented SPAD index, shoot and root fresh weight of cucumber and maize seedlings. SPAD index is a parameter that correlates to chlorophyll content in the leaf tissue. Plants grown without P(-P) and with bulk FePO_4_ (-P + b) as source of P showed higher SPAD index values than the positive control. This effect is a known P deficiency symptom, due to a reduced leaf growth.

Cucumber plants grown with FePO_4_ NPs as P source (-P + NPs) did not present differences from the positive control (Fig. 6a). Regarding shoot biomass production, plants grown with FePO_4_ NPs as P source (-P + NPs) did not show differences from the positive control, while plants grown with bulk FePO_4_ (-P + b) as source of P reached an intermediate biomass value (Fig. 6b). Even if not statistically significant, this trend displayed also for the number of leaves (Fig. 6d). In fact, the values for control plants and plants grown with FePO_4_ NPs as P source (-P + NPs) appear to be higher than the values of other conditions. Considering root biomass production, it is noticeable that cucumber plants grown with FePO_4_ NPs as P source (-P + NPs) showed values significantly higher than control plants (C) (Fig. 6b). Given that a typical response of plants to P deficiency is the alteration of the shoot/root ratio in favor to larger roots growth^46^, this parameter was also evaluated. Figure 6c shows that shoot/root ratio was significantly lower than the positive control for plants grown with both forms of FePO_4_ (NPs and bulk), when applied as sources of P. However, plants grown with FePO_4_ NPs as P source (-P + NPs) have higher values than plants grown with bulk FePO_4_ (-P + b) as source of P.

Regarding the cucumber shoot P contents, we observed a marked reduction in all conditions relative to their positive control (Fig. 6e). However, P contents of plants grown with FePO_4_ NPs, are significantly higher than in plants grown with bulk FePO_4_ as P source and of those grown without the macronutrient. At root level, P concentrations of plants grown with the two forms of FePO_4_ were higher than in plants grown without the macronutrient but not significantly different among them (Fig. 6e).

As far as Fe nutrition is concerned, cucumber plants grown without Fe (-Fe) had significant lower values than other conditions regarding SPAD index (Fig. 6g), shoot and root biomasses (Fig. 6h) and number of leaves (Fig. 6j). Moreover, plants grown without Fe (-Fe) had significant higher shoot/root ratio values than other conditions, reflecting an alteration of biomass allocation condition of Fe deficiency (Fig. 6i). On the other hand, plants grown with FePO_4_ NPs (-Fe + NPs) and bulk FePO_4_ (-Fe + b) as Fe source did not show significant differences from the positive control (C) in all these parameters. Cucumber shoot Fe concentration of plants grown with both forms of FePO_4_ as a source of Fe was not significantly different from the positive control (Fig. 6l). However, Fe concentrations for plants grown with bulk FePO_4_ were slightly lower, even if not significantly. As expected, the shoot Fe content of plants grown without the micronutrient showed the lowest values. Concerning root Fe contents, our results underlined a higher amount of this element in plants grown with FePO_4_ NPs, with levels about three times higher than the plants grown with bulk FePO_4_ and almost nine times higher than the positive control (Fig. 6l).

Regarding the effects on P nutrition in maize, little variations occurred among the experimental treatments when considering fresh biomasses (Fig. 7b). Only the shoots of plants grown without P (-P) were affected by the growth conditions with a significant decrease of the fresh weight. No differences were recorded for the root. However, when considering shoot/root ratio (Fig. 7c), a clear reduction occurs for plants grown without P (-P) and plants grown with bulk FePO_4_ (-P + b) as source of P. On the other hand, plants grown with FePO_4_ NPs as P source (-P + NPs) have values similar to the positive control (Fig. 7c). Regarding SPAD index, differences between -P + NPs and -P + b were recorded with a higher level in the second condition (Fig. 7a). The number of leaves is another parameter that was slightly affected, with a significant variation for –P plants only (Fig. 7d).

Phosphorous content was markedly reduced in maize shoots of plants grown in all treatment conditions in comparison to the positive control (Fig. 7e). However, at the root level maize plants grown with FePO_4_ NPs as P source (-P + NPs), exhibited higher P values than plants grown with bulk FePO_4_ as source of this macronutrient (-P + bulk FePO_4_).

As expected, the SPAD index of maize Fe-deficient plants (-Fe) was dramatically lower than that of control plants. Conversely, plants grown with FePO_4_ NPs as Fe source (-Fe + NPs) showed SPAD values close to those of positive control, and higher than those of plants grown with bulk FePO_4_ as source of Fe (-Fe + b) (Fig. 7g). A similar trend is observable also for the number of leaves (Fig. 7j). Regarding shoot biomass, maize plants grown with FePO_4_ NPs as Fe source (-Fe + NPs) did not show differences from the positive control, while plants grown with bulk FePO_4_ (-Fe + b) had an intermediate biomass value. On the other hand only the root biomass of Fe deficient (-Fe) plants was significantly lower than those grown in the other nutritional conditions (Fig. 7h). Looking at the shoot/root ratio we observe that –Fe + NPs plants have values similar to the control, while -Fe + b plants have values near to the ones of -Fe plants (Fig. 7i).

Maize shoot Fe levels of plants grown without the micronutrient and with bulk FePO_4_ (-Fe + bulk FePO_4_) were respectively 60% and 50% lower than the positive control (Fig. 7l). This reduction is less pronounced when plants were grown with FePO_4_ NPs as Fe source (-Fe + NPs), with a decrease of 35%. Looking at the concentrations of this micronutrient in roots, it appeared remarkable such a high level of this nutrient for plants grown with FePO_4_ NPs (-Fe + NPs), with values even higher than that of the positive control. On the contrary, plants grown with bulk FePO_4_ (-Fe + bulk FePO_4_) showed values similar to those of plants grown without Fe (-Fe).

## Discussion

### Continuous synthesis and citrate capping

The first part of this work was focused on the optimization of a simple, cheap and industrially scalable synthesis method for producing FePO_4_ NPs, which could provide a product with a convenient shelf-life making it potentially exploitable in the fertilizer industry. Several techniques have been proposed for NPs synthesis^26^; among them, co-precipitation appears to be a cheap and easy to use method^27^ and the present work focused on this principle.

However, co-precipitation methods for NPs synthesis used till now in plant nutrition applications are always small-scale batch methods. On the other hand, continuous-flow processes display interesting advantages over batch ones. For example, they can be automated with far less effort than batch reactions, and comprise of a smaller reaction volume, leading to safety benefits if using hazardous reagents or products. Moreover, continuous-flow processes are easy to scale up, simply increasing the operating flow together with the reaction chamber size, maintaining so the residence time unchanged^47,48^. For all these reasons a continuous-flow method was investigated, and a pilot plant for the continuous flow of FePO_4_ NPs synthesis was set up. The system had a productivity of approximately 140 g FePO_4_ NPs·h^−1^, with a size peak of 78 nm, and about 64% of the particles smaller than 100 nm (Fig. 2). Considering these results, this method can be defined simple, economically advantageous and industrially scalable. On the other hand, the particles were not stable, aggregating and sedimenting over time (Fig. 3a). This behaviour, that represents a disadvantage for an industrial application, was solved by introducing a citrate capping treatment - after dialysis, see below - that stabilized the suspension for at least 8 months (Fig. 4b). FT-IR analysis before and after citrate capping proved the adsorption of citrate molecules on particles surface and the measurement of zeta potential indicates that the stabilization is of electrostatic nature (Fig. 5), due to the repulsion of negative charges of carboxylic groups^36^.

The purpose of developing a stable product drove the optimization of particles purification with dialysis, that demonstrated to be a mild purification technique, limiting NPs aggregation (Fig. 2) if compared to centrifugation (Fig. 1). This need also promoted the development of the stabilization with citrate. Comparing the purification method used by us, with others described in the literature, it is evident that the production of NPs is related to their application. For example, in the synthesis of FePO_4_ NPs for the production of more efficient LiFePO_4_ cathodes for batteries^32–35^ no stabilization of NPs was performed. Furthermore, purification methods that were applied (such as filtration and washing with deionized water followed by drying), clearly caused a strong NPs aggregation, as shown by TEM images reported in the above cited papers. However, in these applications the aggregation of NPs is not a concern. On the other hand, the capping of NPs in order to avoid, reduce, or delay aggregation is a practice that is commonly used when NPs are used in suspension in an aqueous medium^37,38^.

In the work published by Liu and Lal^19^, hydroxyapatite (HA) NPs (Ca_5_(PO_4_)_3_OH) were capped with carboxymethylcellulose (CMC), which was added directly in the Ca^2+^ source solution preventing NPs sedimentation. NPs stabilization can also be performed on purified NPs, after the reaction, as we did. Kotsmar *et al*.^49^ for example, stabilized Fe_2_O_3_ NPs with citrate with relatively long time (90 min) and high temperature (90 °C). An advantage of our method of citrate capping is the short time that was needed for the capping treatment (2 minutes), and the execution at room temperature. Furthermore, the citrate-capped NPs were stable even after a 8-month storage at room temperature (24 ± 6 °C).

However, in both methods NPs are never dried, but always kept in liquid suspension, reducing potential health hazards concerned with nanopowders manipulation for both industry workers and farmers.

### FePO_4_ NPs were tested for the effectiveness as source of P and Fe on cucumber and maize plants, grown in hydroponics

To the best of our knowledge, experiments aimed at assessing the effect of FePO_4_ NPs on plant growth are not present in the literature. Liu and Lal (2014) demonstrated that HA NPs (Ca_5_(PO_4_)_3_OH) increased by 30% the growth of soybean cultivated in pot on a solid substrate (peat and perlite) compared to the plants treated with a conventional fertilizer. Our data showed that in cucumber, FePO_4_ NPs caused a higher accumulation of shoot fresh biomass (Fig. 6b) than in deficient plants (-P). On the other hand, FePO_4_ NPs caused a significantly higher shoot growth relatively to bulk FePO_4_ only when used as source of P (-P + NPs). Conversely, in maize, FePO_4_ NPs induced a better growth of shoot than bulk FePO_4_ only when applied as source of Fe (Fig. 7b). Chlorophyll content in leaves is an indicator of the nutritional status of plants. Fe-deficiency generally causes a drastic decrease in chlorophyll content called chlorosis^50^. On the contrary P-deficiency causes a reduced leaf growth, with the consequent higher chlorophyll concentration^51^ together with a purpling of leaves, due to the concomitant anthocyanins accumulation. SPAD index values, evidenced in cucumber (Fig. 6a) a darkening of leaves in plants treated with bulk FePO_4_ as P source (-P + b) comparable to those of plants grown without P (-P). On the contrary plants treated with FePO_4_ NPs (-P + NPs) had SPAD values similar of those measured in control plants. These results suggest that FePO_4_ NPs are an efficient source of P for cucumber in our experimental conditions. However, the same behaviour was not observed in maize possibly indicating that genetically-based differences in root physiology^45^ could play a role in the interaction among plants and NPs. Furthermore, it has to be recalled that also the developmental stage might play a role in the observed behaviour. In maize, the P reservoir in seeds could satisfy plant P needs thus masking the response of the plants at the shoot level. The different behavior in relation to P nutrition between cucumber and maize is confirmed by P concentration data in shoot tissues. In fact, FePO_4_ NPs determined a P concentration in cucumber shoots significantly higher than bulk FePO_4_ (Fig. 6e), while in maize, not significant differences were observed between the two treatments (Fig. 7e).

Plants grown in the absence of Fe showed evident symptoms of deficiency irrespective of the species. However, in the case of cucumber Fe supplied by both forms (NPs and bulk) appeared to be available to the same extent of Fe-EDTA (Fig. 6a–c). On the other hand, in maize FePO_4_ NPs appeared to be more available than bulk FePO_4_. These data were in agreement with the Fe concentrations determined in shoot in both plant species (Figs 6l and 7l).

An effect similar to that of Fe-chelates in preventing Fe chlorosis was reported also in chickpea plants treated with FeCO_3_ NPs^18^, and peanut plants treated with Fe_2_O_3_ NPs^15^. Moreover, similarly to what here shown with cucumber, no differences in SPAD index values between plants grown with Fe_2_O_3_ NPs and bulk Fe_2_O_3_ were observed in soybean^17^. The above mentioned species are both Strategy I species for Fe acquisition, basing the strategy on rhizosphere acidification, carboxylic acids extrusion and the reduction of Fe(III) to Fe(II). Differently, our experiment with maize indicated that FePO_4_ NPs are an efficient Fe source for this species as suggested by SPAD index values (Fig. 7g). These differences could reasonably be based on the ability of strategy II plants to excrete in Fe-shortage condition chelating molecules (phytosiderophores) characterized by high stability constant for metals^52^.

The effect of the treatments on the root apparatus was not particularly evident on fresh weight of both plant species. No significant differences on root dry weight were observed in pot experiments when Fe_2_O_3_ NPs were applied to soybean^17^ and peanuts^15^ plants. On the other hand, Fig. 6b shows an interesting effect on cucumber root fresh weight. In fact, NPs exerted a positive effect on these parameters that can be tentatively explained as a biostimulant-like effect^53^. Positive effects of nanomaterials, in particular carbon nanomaterials (CNMs), on root plant growth are reported^54^. The treatments with multi-wall carbon nanomaterials (MWCNTs) increased the root length of hydroponically grown ryegrass plants^55^ and of corn seedlings in agar medium^56^. Similar results were observed when onion and cucumber plants grown in hydroponics were exposed to uncoated CNT^57^. Therefore, due to the diverse origin of the above-mentioned nanomaterials and the different species of plants it is reasonable to hypothesize that size of NPs is the effector of the enhanced root growth.

Concerning P concentration in root tissue, we could observe that in the case of maize differences between NPs- and bulk-treated plants were present (Fig. 7e). The higher P concentration in NPs-fed roots suggests that even if at the shoot level no significant symptoms of P deficiency were recordable, at the root level NPs could counteract an initial condition of P shortage, that is recordable for plants treated with bulk FePO_4_.

Considering Fe concentration in cucumber roots, it can be noticed that this element accumulates in amounts four times higher than in positive control when supplied as bulk FePO_4_, and even nine times higher when supplied as FePO_4_ NPs (Fig. 6l). Reported values are relative to total Fe, and we cannot discriminate between symplastic and apoplastic Fe. However on the bases of these results we could assume that NPs suspended in nutrient solution can be adsorbed on the root surface and Fe somehow utilized by plants. Regarding Fe concentration in maize roots, plants grown with bulk FePO_4_ do not differ significantly from Fe-deficient plants with levels about 3 times lower than control plants. Interestingly the treatment with FePO_4_ NPs determined values that are double than the control (Fig. 7l) thus confirming the NPs ability to improve the nutrient delivery. This result is also coherent with what observed with SPAD index (Fig. 7g). Moreover, Kulikova *et al*. (2017)^58^, studied the availability of nano-sized Fe hydroxides stabilized with humic substances on wheat seedlings, and showed, similarly to us, that plants grown with this form of Fe hydroxides accumulated in roots from 4 to 10 times more Fe than control plants, even if in shoots the concentrations were comparable.

In conclusion, FePO_4_ NPs showed to be an effective source of P and Fe, particularly if compared to the non-nano counterpart. However, the treatment with NPs caused differential responses in cucumber and maize (Figs 6 and 7). This could be explained not only in term of physiological differences between the two species but also on the bases of the difference in the timing of the response to the deficiencies that the plants displayed in our experimental conditions. These results represent an encouraging starting point for further research, aimed to determine with accuracy the effects of a multi-nutrient nanofertilizer on different plant species both in hydroponic and in a real plant-soil microcosm.

## Material and Methods

In order to find a simple, economically advantageous, and industrially scalable method for the synthesis of iron (III) phosphate (FePO_4_) NPs many synthesis methods were assayed. All used methods are based on co-precipitation: a kind of reaction where two solutions of soluble salts are mixed together and an insoluble product is formed, typically a salt with a low solubility product. FePO_4_, due to its low solubility product (1.3·10^−22^ for FePO_4_ and 9.91·10^−16^ for FePO_4_·2H_2_O) is commonly synthesized with this strategy.

### Rapid mixing-based synthesis

A continuous-flow synthesis method for FePO_4_ NPs was set up on the bases of Zhang *et al*. (2014) with minor modifications. This method is based on the extremely fine and rapid mixing of two solutions in a mixing chamber due to a low residence time in the chamber obtained through high flows and a small volume of it. It consisted in two dosing pumps (EMEC model AMS MF 2505 V) for solutions pumping, and an HPLC mixing tee as mixing chamber (dead volume 10 µL). The system can operate with a flow of each solution of 7.5 L·h^−1^, with solution “A” containing 0.1 M FeCl_3_ and 0.02 M H_3_PO_4_, and a solution “B” containing 0.1 M K_2_HPO_4_ at pH 9.10, for a potential productivity of 15 l·h^−1^ of raw FePO_4_ NPs suspension.

Half of the suspension was purified through three steps of centrifugation at 4500 rcf for 15 min and resuspension in deionized water.

The other half suspension was purified through dialysis with deionized water for 24 hours. The retentate:water ratio was 1:40, and the water was changed four times at increasing time intervals. The Molecular Weight Cut-Off (MWCO) of the membrane was 14 kDa.

### Laboratory-scale batch synthesis for hydroponic trials

Twenty-five millilitres of a 0.6 M K_2_HPO_4_ solution were added drop by drop to 25 mL of a solution containing 0.6 M Fe(NO_3_)_3_ under continuous stirring at 600 rpm at room temperature (25 °C). After the forming of a slight turbidity the solution was centrifuged 20 min at 4500 rcf, and the small resulting pellet was discarded. The clear supernatant was treated at 85 °C for 1 h in order to form NPs, and then let cool to room temperature under continuous stirring at 600 rpm. The obtained NPs suspension was then purified from by-products through dialysis with deionized water for 24 hours. The retentate:water ratio was 1:40, and the water was changed four times, at increasing time intervals. The Molecular Weight Cut-Off (MWCO) of the membrane was 14 kDa.

### Citrate capping of NPs

Fifty millilitres of the suspension were citrate-capped through the adding of 5.55 mL of 1 M tribasic potassium citrate and thorough vortexing for 2 min. The suspension was then immediately purified from the excess of citrate through dialysis.

### Size distribution of NPs and Zeta Potential

Size distribution and Zeta Potential were determined through DLS analysis with a Malvern Zetasizer (ZS instrument) operating with a He-Ne laser at 633 nm. Samples for DLS analysis were diluted 1:20 in deionized water and analyzed measuring 173° backscatter. Sizes distribution were chosen to be shown in number, following the definition set by the European Commission: “…material containing particles, in an unbound state or as an aggregate or as an agglomerate and where, for 50% or more of the particles in the number size distribution, one or more external dimensions is in the size range 1 to 100 nm”^58^.

### TEM observation of FePO_4_ NPs

Ten microliters of FePO_4_ NPs suspension were deposited on copper (Cu) grids and let dry at room temperature. NPs samples were observed with a Tecnai G2 (FEI) Transmission Electronic Microscope (TEM), operating at 120 kV.

### Fe and P quantification in FePO_4_ NPs suspension

The obtained suspensions were prepared for Fe and P quantification dissolving 2.5 mL of NPs suspension with 2.5 mL of concentrated HCl (37%). The obtained solution was then diluted in order to fit into the range of calibration curves of the quantification methods. Iron was quantified with the method of Stookey^59^ using PDT disulfonate as chromophore and P concentration was determined with the method of Riley and Murphy^60^.

### X-Ray diffraction analysis

Samples structure was analyzed by means of X-ray diffraction (XRD) with a Thermo ARL X’TRA powder diffractometer, operating in the Bragg-Brentano geometry equipped with a anode of Cu Kα radiation (λ = 1.5418 Å) and using a Peltier Si(Li) cooled solid state detector. The diffraction pattern was recorded over the 5°–90° angle range with a step-size of 2θ = 0.03°. Samples suspensions were dryed at 90 °C for 3 days and then carefully homogenized with a mortar. The so-obtained powders were suspended in few drops of ethanol and deposited onto a low background sample stage. Ethanol was left to evaporate before starting the analysis.

### FT-IR spectroscopy

Vibrational characterization of both bare and capped FePO_4_ nano-powder was carried out by means of absorption spectroscopy measurements in the mid infrared (MIR) region. In fact, chemically bonded ions (*i.e.*: dipoles) vibrate at characteristic frequencies, and when exposed to infrared radiation, they absorb the radiation at frequencies that match their vibrational modes. Measuring the radiation absorption as a function of frequency gives a spectrum that can be used to identify functional groups and compounds.

FT-IR absorbance spectra in the 4000–400 cm^−1^ range were collected at room temperature with a resolution of 4 cm^−1^ using a JASCO spectrometer (model FT/IR-660 plus) equipped with a Tri-Glycine-Sulfate (TGS) detector, set in transmission configuration. To this aim the iron phosphate powders obtained for XRD analysis were dispersed into KBr pellets at a ratio of 1 mg powders: 300 mg KBr. In order to allow for a better comparison between different samples, a proper baseline has been carefully subtracted from each FT-IR spectrum.

### Plant material and growth conditions

*Cucumis sativus* var. Viridis F1 hybrid seeds (FranchiSementi S.p.A.) were germinated on paper towel moistened with 1 mM CaSO_4_ at 24 °C in the dark. After 6 days, 6 seedlings per condition were transferred to 2-L pots containing aerated nutrient solution. *Zea mays* L. inbred line P0423 seeds (Pioneer Hybrid Italia S.p.A.) were germinated on paper towel moistened with deionized water at 25 °C in the dark. After 3 days, 6 seedlings per condition were transferred to 2-L pots containing aerated nutrient solution. Plants were grown under a 16/8 h light/dark photoperiod at 24 ± 2 °C with a light intensity of 200–250 μmol m^−2^ s^−1^ as PAR (Photosynthetically Active Radiation) at the plant level. The complete nutrient solution (control) was a modified Hoagland solution^61^ with the following composition: 0.7 mM K_2_SO_4_, 2 mM Ca(NO_3_)_2_, 0.5 mM MgSO_4_, 0.1 mM KH_2_PO_4_, 0.1 mM KCl, 100 μM FeNaEDTA, 10 μM H_3_BO_3_, 0.5 μM MnSO_4_, 0.5 μM ZnSO_4_, 0.2 μM CuSO_4_ and 0.01 μM (NH_4_)_6_Mo_7_O_24_. Ten experimental conditions were set up: plants grown in a complete nutrient solution (C), plants grown without P (-P), plants grown without Fe (-Fe), plants grown without both P and Fe (-P-Fe), plants grown with FePO_4_ NPs as source of P (-P + NPs), plants grown with FePO_4_ NPs as source of Fe (-Fe + NPs), plants grown with FePO_4_ NPs as source of both P and Fe (-P-Fe + NPs), plants grown with bulk FePO_4_ as source of P (-P + bulk FePO_4_), plants grown with bulk FePO_4_ as source of Fe (-Fe + bulk FePO_4_), and plants grown with bulk FePO_4_ as source of both P and Fe (-P-Fe + bulk FePO_4_). Both FePO_4_ NPs and bulk FePO_4_ (Sigma-Aldrich, 436011) were added at a concentration equivalent to 100 µM. In the solutions without KH_2_PO_4_, K^+^ cations were balanced using 0.2 mM KCl instead of 0.1 mM. The nutrient solution was changed twice a week and adjusted to pH 6 with 1 N NaOH. Three growth and treatment experiments (biological replicates) with six plants each (technical replicates) were independently repeated, for a total of 18 plants.

### SPAD index measurement and plants sampling

The sampling-time points for both plant species were chosen on observational basis, looking for appreciable differences in symptoms among the different experimental conditions. Following this rationale, the plant sampling occurred after 14 and 17 days of growth for cucumber and maize, respectively. SPAD Index was determined taking five measurements per leaf using a SPAD-502 Plus Chlorophyll meter^®^ (Konica Minolta). In the case of cucumber, at the end of the experiment (14 days) the SPAD index was determined for the first (the youngest fully expanded) leaf of each plant, that exhibited more differences in SPAD index, so this parameter was chosen to be shown instead of the mean SPAD index of all leaves, value that was used for maize (17 days).

### P and Fe content determination in plant tissues

Plant tissues of three plants per condition were weighted and dried for 72 hours at 60 °C. Then, dried samples were weighted and milled using mortar and pestle, and then approximately 10 to 20 mg of homogenized material was mineralized with 250 μL of ultra-pure 68% HNO_3_ (Romil LTD) and 1 mL of 30% H_2_O_2_ at 180 °C for 20 minutes in a StartD^®^ microwave digestion system (Milestone Srl). The digested samples were diluted up to 2% HNO_3_ with ultra-pure grade water (18.2 MΩ·cm at 25 °C), and then analyzed using an Agilent 7500ce ICP-MS detection system (Agilent technologies).

## Supplementary information

Dataset 1

## Data Availability

All data generated or analysed during this study are included in this published article (and its Supplementary Information files).
