# Supplementary material for: FePO4 nanoparticles produced by an industrially scalable continuous-flow method are an available form of P and Fe for cucumber and maize plants
Source: Sci Rep. 2019 Aug 2;9:11252. doi: 10.1038/s41598-019-47492-y (PMC6677738; doi:10.1038/s41598-019-47492-y)
Supplement: Supplementary file 1 — Dataset 1 [file 41598_2019_47492_MOESM1_ESM.docx]

**FePO_4_ nanoparticles produced by an industrially scalable continuous-flow method are an available form of P and Fe for cucumber and maize plants**

**Davide Sega^1^, Giuseppe Ciuffreda^2^, Gino Mariotto^3^ Barbara Baldan^4^, Anita Zamboni^1^, Zeno Varanini^1^**

^1^ Department of Biotechnology, University of Verona, Italy

^2^ Fabbrica Cooperativa Perfosfati Cerea, Cerea (VR), Italy

^3^Department of Computer Science, University of Verona, Italy

^4^ Department of Biology, University of Padua, Italy


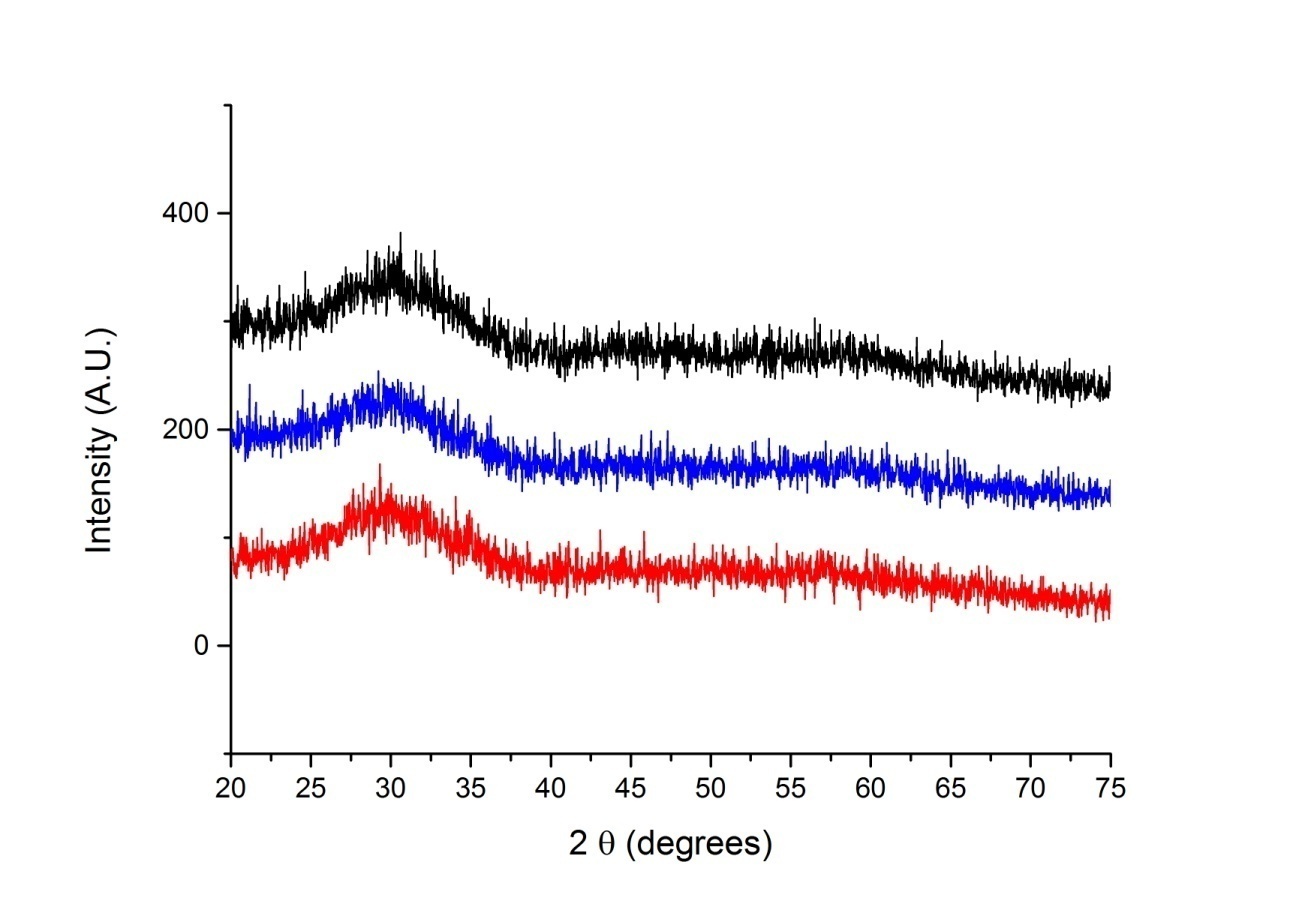


Supplementary figure S1| Superimposed X-Ray Diffraction spectra of bulk FePO_4_ (black line), bare FePO_4_ NPs (blue line), citrate-capped FePO_4_ NPs (red line).

**Supplementary table S1|** Comparison of cucumber plants treated with different nutrient sources. Data are means ± SD of three independent experiments with six plants (technical replicates) each (One-way ANOVA with Turkey’s post hoc test, p<0.05).

|  | **C** | **-P** | **-P+NPs** | **-P+b** |
| --- | --- | --- | --- | --- |
| **SPAD** | 42.2±3.0b | 55.3±3.0a | 44.3±1.7b | 52.6±3.1a |
| **Shoot FW (g)** | 2.73±0.73a | 1.18±0.17c | 2.33±0.54a | 1.62±0.36b |
| **Root FW (g)** | 1.49±0.55b | 1.52±0.23b | 2.14±0.52a | 1.85±0.42ab |
| **S/R (FW)** | 1.92±0.43a | 0.78±0.10c | 1.14±0.32b | 0.88±0.10c |
| **Number of Leaves** | 2.00±0.34a | 1.61±0.92a | 2.17±0.71a | 1.78±0.94a |
| **P shoot (µg/gFW)** | 1315±90.7a | 184±34.3c | 514±98.3b | 233±14.4c |
| **P root (µg/gFW)** | 230.0±24.46a | 55.16±5.30c | 153.7±45.62b | 136.9±53.34b |
| **Fe shoot (µg/gFW)** | 16.78±3.59a | 21.39±7.53a | 17.19±8.97a | 23.90±5.18a |
| **Fe root (µg/gFW)** | 33.24±6.25b | 34.03±6.77b | 234.4±99.8a | 174.3±68.8a |
|  | **C** | **-Fe** | **-Fe+NPs** | **-Fe+b** |
| **SPAD** | 42.2±3.0a | 18.1±13.0b | 39.6±3.2a | 41.2±3.7a |
| **Shoot FW (g)** | 2.73±0.73a | 1.62±0.60b | 2.88±0.71a | 2.83±0.64a |
| **Root FW (g)** | 1.49±0.55a | 0.71±0.38b | 1.68±0.35a | 1.56±0.45a |
| **S/R (FW)** | 1.92±0.43b | 2.53±0.68a | 1.74±0.34b | 1.86±0.30b |
| **Number of Leaves** | 2.00±0.34ab | 1.67±0.49b | 2.28±0.57a | 2.31±0.48a |
| **P shoot (µg/gFW)** | 1315±90.7a | 937±96.1b | 1305±58.9a | 1276±175a |
| **P root (µg/gFW)** | 230.0±24.46b | 223.1±32.76b | 336.9±108.3a | 233.4±111.9b |
| **Fe shoot (µg/gFW)** | 16.78±3.59a | 7.29±2.50b | 18.14±5.69a | 14.74±4.65a |
| **Fe root (µg/gFW)** | 33.24±6.25bc | 3.86±2.45c | 289.9±97.0a | 106.7±30.1b |
|  | **C** | **-P-Fe** | **-P-Fe+NPs** | **-P-Fe+b** |
| **SPAD** | 42.2±3.0b | 28.6±8.3c | 43.5±3.8b | 52.1±3.5a |
| **Shoot FW (g)** | 2.73±0.73a | 1.22±0.42b | 2.28±0.68a | 1.54±0.27b |
| **Root FW (g)** | 1.49±0.55b | 0.99±0.57c | 2.09±0.59a | 1.87±0.41ab |
| **S/R (FW)** | 1.94±0.43a | 1.57±0.76a | 1.10±0.19b | 0.84±0.11b |
| **Number of Leaves** | 2.00±0.34a | 1.39±0.61b | 1.89±0.90ab | 1.44±0.62ab |
| **P shoot (µg/gFW)** | 1315±90.7a | 208±24.2c | 387±64.4b | 198±16.6c |
| **P root (µg/gFW)** | 230.0±24.46a | 94.08±18.25c | 177.5±34.68b | 114.9±14.40c |
| **Fe shoot (µg/gFW)** | 16.78±3.59a | 8.76±3.26b | 11.39±2.73b | 11.73±2.30b |
| **Fe root (µg/gFW)** | 33.24±6.25bc | 3.36±1.07c | 214.7±95.3a | 96.1±24.9 |

**Supplementary table S2|** Comparison of maize plants treated with different nutrient sources.Data are means ± SD of three independent experiments with six plants (technical replicates) each (One-way ANOVA with Turkey’s post hoc test, p<0.05).

|  | **C** | **-P** | **-P+NPs** | **-P+b** |
| --- | --- | --- | --- | --- |
| **SPAD** | 41.2±2.7ab | 39.7±1.9b | 42.0±2.0a | 39.2±2.6b |
| **Shoot FW (g)** | 6.04±2.54a | 4.27±1.00b | 5.85±0.72a | 5.17±1.44ab |
| **Root FW (g)** | 1.34±0.43a | 1.65±0.69a | 1.48±0.32a | 1.50±0.39a |
| **S/R (FW)** | 4.43±0.92a | 2.78±0.62c | 4.09±0.61ab | 3.45±0.55b |
| **Number of Leaves** | 4.88±0.33a | 4.17±0.38b | 4.67±0.59a | 4.39±0.50ab |
| **P shoot (µg/gFW)** | 825±16.0a | 157±30.0b | 237±32.0b | 173±35.6b |
| **P root (µg/gFW)** | 155±33.0a | 59.1±14.6c | 128±23.4a | 92.8±24.7b |
| **Fe shoot (µg/gFW)** | 9.79±0.91b | 11.09±2.00b | 17.70±4.74a | 14.38±5.34ab |
| **Fe root (µg/gFW)** | 42.83±18.06c | 34.83±7.00c | 269.10±58.39a | 105.9±55.86b |
|  | **C** | **-Fe** | **-Fe+NPs** | **-Fe+b** |
| **SPAD** | 41.2±2.7a | 11.4±2.8d | 36.8±2.5b | 24.2±2.7c |
| **Shoot FW (g)** | 6.04±2.54a | 2.45±0.61c | 6.56+2.23a | 4.39±1.26b |
| **Root FW (g)** | 1.34±0.43ab | 1.02±0.31b | 1.44±0.29a | 1.44±0.31a |
| **S/R (FW)** | 4.43±0.92a | 2.50±0.77b | 4.55±1.13a | 3.07±0.71b |
| **Number of Leaves** | 4.88±0.33a | 3.44±0.51c | 4.72±0.57ab | 4.33±0.49b |
| **P shoot (µg/gFW)** | 825±16.0bc | 1227±212a | 616±105d | 778±126cd |
| **P root (µg/gFW)** | 155±33.0b | 225±45.1a | 160±22.1b | 109±47.7ab |
| **Fe shoot (µg/gFW)** | 9.79±0.91a | 3.94±1.05c | 6.22±0.76b | 5.19±0.99b |
| **Fe root (µg/gFW)** | 42.83±18.06b | 2.80±1.78c | 87.08±29.41a | 14.50±5.24c |
|  | **C** | **-P-Fe** | **-P-Fe+NPs** | **-P-Fe+b** |
| **SPAD** | 41.2±2.7a | 14.9±3.8c | 40.3±2.1a | 35.8±2.3b |
| **Shoot FW (g)** | 6.04±2.54a | 2.40±0.83c | 5.40±0.97ab | 4.34±1.32b |
| **Root FW (g)** | 1.34±0.43a | 1.11±0.42a | 1.39±0.34a | 1.27±0.28a |
| **S/R (FW)** | 4.43±0.92a | 2.25±0.53c | 3.97±0.63ab | 3.38±0.70b |
| **Number of Leaves** | 4.88±0.33a | 3.39±0.50d | 4.50±0.51b | 4.06±0.24c |
| **P shoot (µg/gFW)** | 825±16.0a | 0.229±35.4b | 230±35.6b | 171±54.6b |
| **P root (µg/gFW)** | 155±33.0a | 59.3±12.6c | 98.2±11.4b | 86.8±20.5bc |
| **Fe shoot (µg/gFW)** | 9.79±0.91a | 3.35±1.24b | 8.42±3.91a | 7.22±0.58b |
| **Fe root (µg/gFW)** | 42.83±18.06b | 2.20±1.50c | 123.50±21.28a | 35.14±55.78b |
